# Costs of dementia: Comprehensive Estimates from U.S. Cost of Dementia Model (USCDM)

**DOI:** 10.1002/alz70860_101358

**Published:** 2025-12-23

**Authors:** Hanke Heun‐Johnson, Bryan Tysinger, Johanna Thunell, Duncan Ermini Leaf, Geoffrey Joyce, Mireille Jacobson, Manying Cui, Sidra Haye, Dana Goldman, Julie M Zissimopoulos

**Affiliations:** ^1^ University of Southern California, Los Angeles, CA, USA

## Abstract

**Background:**

Dementia is associated with substantial economic burden for individuals, their care partners and family, communities, and society. There are myriad types of costs including those associated with medical care, long term care, supportive services, quality of life as well as others such as vulnerability to exploitation. There is a gap in annual, nationally representative, comprehensive cost estimates and variability across types of costs and persons. Tracking these costs over time as prevention, treatment and policy evolve will support efforts to reduce the economic impact of dementia.

**Method:**

We used nationally representative data from the Health and Retirement Study (HRS) of U.S. adults over age 50 and other nationally representative data sources. We employed the U.S. Cost of Dementia Model (USCDM) ‐ a dynamic microsimulation model and estimated medical and long term care costs, costs of time and earnings loss associated with unpaid family caregiving and quality of life of persons living with dementia and their care partners.

**Result:**

In 2025, an estimated 5.6 million people are living with dementia in the United States. The medical and long‐term care costs for dementia total $232 billion. Families and friends provide 6.8 billion hours of care, which is worth $233 billion. Many care partners also reduce their work hours or leave their jobs entirely, leading to a loss of $8.2 billion in annual earnings among care partners of parents with dementia. In addition to financial costs, people with dementia and their care partners experience a significant loss in quality of life, valued at $302 billion for those living with dementia and $5.9 billion among caregivers of parents or spouses with dementia.

**Conclusion:**

The USCDM, a comprehensive, well‐validated dynamic microsimulation model, provides national, comprehensive estimates of total costs of dementia and by type of costs and who bears them. It is part of a larger effort to build capacity for researchers and policymakers to reduce the future impact of dementia.

